# Small-Scale Plastic Deformation of Nanocrystalline High Entropy Alloy

**DOI:** 10.3390/e20110889

**Published:** 2018-11-20

**Authors:** Sanghita Mridha, Mageshwari Komarasamy, Sanjit Bhowmick, Rajiv S. Mishra, Sundeep Mukherjee

**Affiliations:** 1 Department of Materials Science and Engineering, University of North Texas, Denton, TX 76203, USA; 2 Bruker Nano Surfaces, Minneapolis, MN 55344, USA

**Keywords:** nanocrystalline materials, high entropy alloy, sputtering, deformation and fracture, strain rate sensitivity

## Abstract

High entropy alloys (HEAs) have attracted widespread interest due to their unique properties at many different length-scales. Here, we report the fabrication of nanocrystalline (NC) Al_0.1_CoCrFeNi high entropy alloy and subsequent small-scale plastic deformation behavior via nano-pillar compression tests. Exceptional strength was realized for the NC HEA compared to pure Ni of similar grain sizes. Grain boundary mediated deformation mechanisms led to high strain rate sensitivity of flow stress in the nanocrystalline HEA.

## 1. Introduction

High entropy alloys (HEAs) represent an alloy design paradigm of combining five or more elements in equiatomic or near-equiatomic proportions [1,2]. In certain compositions, high configurational entropy suppresses intermetallic compound formation and leads to single-phase solid solution [3]. HEAs have attracted widespread interest due to their intriguing physical and mechanical properties [3]. Some of the appealing properties include exceptional ductility [3], outstanding thermal stability [4], and cryogenic fracture toughness [5]. Bulk of the research on HEAs have focused on alloy development [3], phase stability [6], and mechanical behavior of coarse grained (CG) and fine-grained systems [3,7,8]. But there are limited reports on nanocrystalline (NC) HEAs and their small-scale deformation behavior [9,10]. NC metals typically show very high strength [11] and good fatigue resistance [12]. Body-centered cubic NbMoTaW refractory NC HEA exhibited exceptional strength at small scales and ductility [9]. Furthermore, NC HEA retained yield strength (YS) of over 5 GPa up to 600 °C denoting an exceptional nano-structural stability [10]. Similar studies for face-centered cubic (FCC) systems could provide insights into their deformation mechanisms at reduced length-scale and pave the way for new application domains towards low-cost, durable, ductile, and strong FCC HEAs.

Al_0.1_CoCrFeNi HEA is a canonical example of a FCC single phase multi-principal element alloy whose mechanical properties have been widely reported. Komarasamy et al. [13] examined the work hardening mechanisms in coarse-grained (CG) (a few mm) and fine-grained (FG) (~3–14 µm) Al_0.1_CoCrFeNi HEA and concluded that both the conditions exhibited deformation twinning mediated plasticity. Following that, Choudhuri et al. [14] investigated the plastic deformation mechanisms of the same alloy in CG and FG conditions using transmission electron microscopy. After quasi-static tensile testing, both the microstructures exhibited nanoscale (~2 nm) twins signifying twinning assisted plastic deformation. Wu et al. [15] also noted a similar behavior with deformation twins in both CG and FG materials. Furthermore, an increase in average twin spacing and a reduction in twin thickness was observed in FG condition as compared with CG material. Kumar et al. [16] examined the high strain rate compression behavior of CG Al_0.1_CoCrFeNi HEA and observed a similar work hardening behavior as quasi-static compression. Yu et al. [17] investigated the deformation mechanism in Al_0.1_CoCrFeNi HEA subjected to high pressure torsion (HPT). Deformation via dislocation slip was noted at low strains while deformation twinning was activated at large plastic strains. Furthermore, a large strain rate sensitivity of 0.035 was obtained signaling grain boundary related deformation mechanism in the HPT condition. In Al_0.1_CoCrFeNi HEA, Feng et al. [18] introduced high density of nano-twins and stacking faults, and investigated the mechanical behavior at small length scales. The pillars exhibited a compressive strength of 4.0 GPa with 15% compressive ductility. The exceptional mechanical properties were attributed to the stability of stacking faults and its effective hindrance to dislocation motion.

In this paper, we report on the nano-mechanical behavior of NC Al_0.1_CoCrFeNi HEA (average grain size ~40 nm) synthesized using magnetron sputtering technique. Stress–strain response was obtained by nano-pillar compression, concurrent with direct observation of their deformation behavior inside a scanning electron microscope (SEM). Different strain rates were used for strain rate sensitivity (*m*) analysis.

## 2. Materials and Methods

Target alloy of composition Al_0.1_CoCrFeNi was prepared using high purity elements (99.99%) and arc melted in an Ar atmosphere. A thin film of the alloy was deposited by magnetron sputtering technique (AJA International, Scituate, MA, USA) on a silicon substrate at room temperature (RT), with the base and process pressure maintained at ~3 × 10^−6^ torr, and ~5 × 10^−3^ torr, respectively. An Ar atmosphere (flow rate 10 sccm) was used and applied power was 75 W. Nano-pillars were synthesized by milling the thin film using Focused Ion Beam (FIB) (FEI). A concentric circular pattern was used to mill out the nano-pillar. Gallium ion beam with current of 10 pA, and operating voltage of 30 kV was used for milling and the pillars had an average diameter of ~450 nm. ImageJ software was used to determine the grain size. In situ compression tests were performed on the nano-pillars with a SEM equipped with PicoIndenter PI 85 (Bruker Nano Surfaces, Minneapolis, MN, USA) using a 2 µm diameter diamond flat punch. Uniaxial compression tests were conducted at strain rates of 1.2 × 10^−1^, 1.9 × 10^−2^, and 7.5 × 10^−3^ s^−1^.

## 3. Results and Discussion

**Figure 1:**
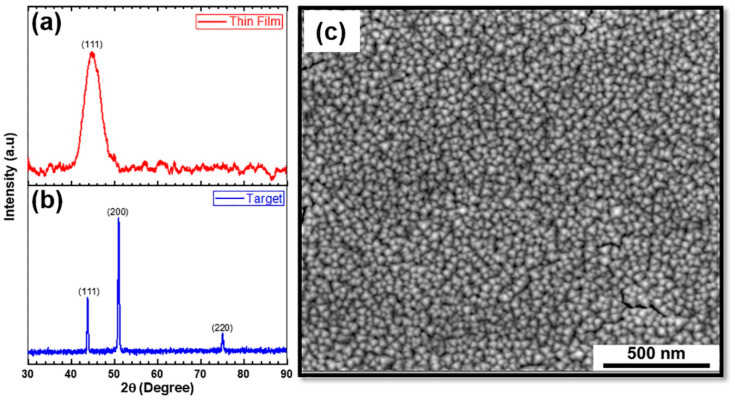
X-Ray diffraction (XRD) patterns of (**a**) Al_0.1_CoCrFeNi thin film and (**b**) Al_0.1_CoCrFeNi target, showing peaks corresponding to the face-centered cubic (FCC) phase; (**c**) high-magnification scanning electron microscope (SEM) image of the thin film showing nano-sized grains with an average grain size of ~40 ± 5 nm.

**Figure 2:**
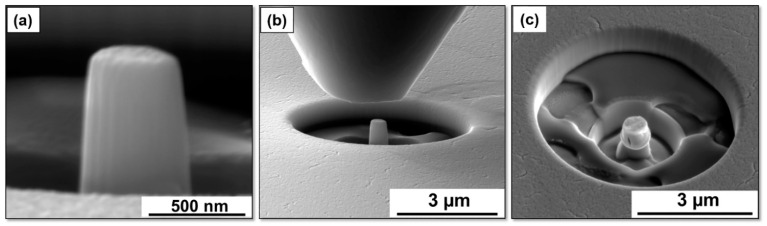
SEM images of the (**a**) nano-pillar with diameter 450 nm (**b**) diamond punch and the nano-pillar, and (**c**) nano-pillar after the compression test.

Figure 1a,b shows the X-ray diffraction (XRD) patterns for Al_0.1_CoCrFeNi HEA thin film and target, respectively, demonstrating a single phase FCC structure. SEM image of the thin film (Figure 1c) shows uniformly distributed nano-sized grains with an average grain size of ~40 ± 5 nm. SEM images of NC nano-pillar HEA before and after compression tests are shown in Figure 2. The pillar diameter was around 450 nm (Figure 2a). Figure 2b shows the nano-pillar with the diamond flat punch just before the compression test. Uniform plastic deformation of the nano-pillar with no evidence of buckling was observed (Figure 2c). Furthermore, crack propagation parallel to the loading direction can be observed in Figure 2c.

**Figure 3:**
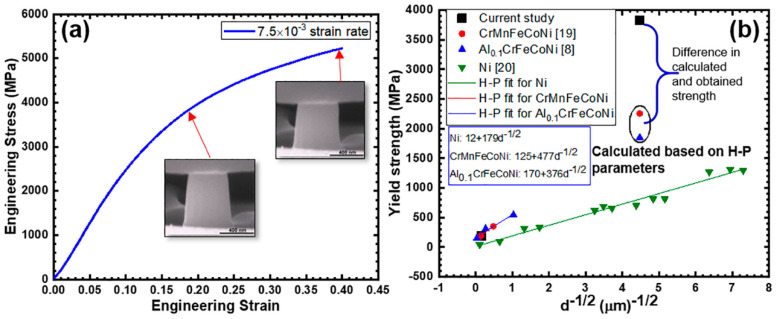
(**a**) Compressive engineering stress–strain curve at strain rate of 7.5 × 10^−3^ s^−1^ showing YS of 3829 MPa. Insets show the *in situ* image of pillar at various stages; (**b**) YS vs. *d*^−1/2^ plot with Hall–Petch equation fit for Ni and various HEAs.

Compressive engineering stress-strain curve at a strain rate of 7.5 × 10^−3^ s^−1^ is shown in Figure 3a. Inset images of the pillar at various deformation intervals clearly show that the plastic deformation was uniform without buckling of the nano-pillar. YS of nano-pillar Al_0.1_CoCrFeNi HEA at 7.5 × 10^−3^ s^−1^ strain rate was ~3829 MPa. YS of the CG material of the same composition was ~190 MPa indicating a 20 fold increase in strength for the NC HEA. Al_0.1_CoCrFeNi forms a single phase alloy with FCC crystal structure without any secondary phases. Therefore, only grain size strengthening contribution was investigated. To that end, YS versus *d*^−1/2^ correlation (where *d* represents grain size) for various HEAs, pure Ni, and the current study are shown in Figure 3b. Hall–Petch relation for various HEAs and Ni are shown in Figure 3b, based on the following equation:

*σ_YS_* = *σ*_o_ + *kd*^−1/2^ (1)

where, *σ*_o_ is the strength of the material for infinitely large grain size also called lattice friction stress, *k* is the Hall-Petch coefficient, and *d* is the grain size. The Hall–Petch equation with coefficients, *σ*_o_ and *k*, for all the conditions are given in the bottom inset of Figure 3b. Hall–Petch coefficients based on two independent HEA investigations were used to calculate the expected grain size strengthening contribution for the current condition with ~50 nm grain size. The calculated YS based on Otto et al.’s [19] and Nilesh et al.’s [8] Hall–Petch coefficients were 2255 and 1849 MPa, respectively. Remarkably, the obtained YS in the current investigation was ~1500 MPa higher than the predicted strength values. This may be attributed to the shift in deformation mode from dislocation-controlled to grain boundary mediated plastic deformation. For the same grain size, HEAs exhibited strength values two fold higher than that of pure Ni [20]. In addition to Hall–Petch and grain boundary mediated deformation mechanism, size effect may also dominate the YS of NC Al_0.1_CoCrFeNi HEA, which is out of the scope of the current paper. In CoCrCuFeNi HEA, Zhang et al. [21] investigated size-dependent YS based on micro-/nano-pillar uniaxial compression tests and noted that the HEA did exhibit size-dependent mechanical properties.

**Figure 4:**
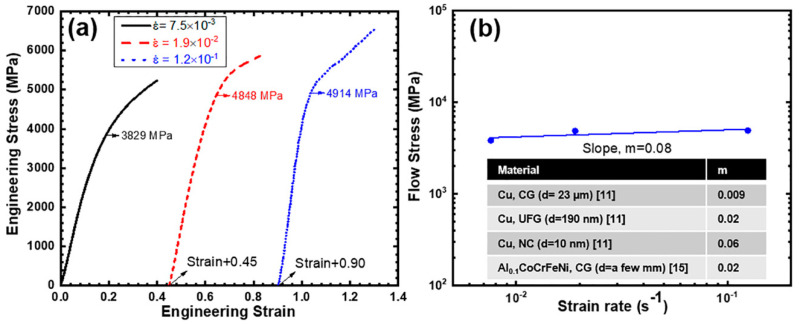
(**a**) Compressive engineering stress-strain plot at different strain rates showing the increase in tensile strength with increase in strain rate; (**b**) flow stress at 1% offset strain versus strain rate plot in logarithmic scale to calculate strain rate sensitivity (*m*).

Effect of strain rate on yield strength and strain rate sensitivity calculation are shown in Figure 4a,b, respectively. Stress-strain curves for 1.9 × 10^−2^ s^−1^ and 1.2 × 10^−1^ s^−1^ strain rates were shifted along the strain axis for a clear representation. With the increase in strain rate, yield strength of the NC nano-pillar Al_0.1_CoCrFeNi HEA increased. This indicates a positive strain rate sensitivity of flow stress at room temperature which was observed in other investigations as well [22]. Strain rate sensitivity (*m*) is defined as:

(2) $$m=\frac{\partial \ln\sigma }{\partial \ln\dot{\varepsilon }}$$

where, σ is the flow stress and $\dot{\varepsilon }$ is the strain rate. Following this equation, slope of 1% flow stress and strain rate in logarithmic scale yielded a strain rate sensitivity of 0.08 as presented in Figure 4b. Comparison with *m* values reported in literature for other materials is given as inset in Figure 4b. As can be clearly seen, *m* value increases with the reduction in grain size. For example, CG and NC copper exhibited *m* values of 0.009 and 0.06, respectively [11]. Furthermore, *m* value of CG HEA was higher than CG conventional metals/alloys, which was attributed to fluctuating lattice energy controlled deformation mechanism. In the current investigation, due to the nano-scale grain size, there is high possibility that dislocation controlled processes were suppressed and grain-boundary mediated processes were activated. Furthermore, *m* value of 0.08 for NC HEA would translate into lower apparent activation volume of dislocation as compared to NC copper with *m* value of 0.06. Therefore, in addition to differences in CG material due to lattice distortion controlled dislocation activity, current results suggest that grain boundary mediated plastic deformation was influenced by the inherent lattice distortion of HEA.

An important implication of the current investigation is the viability of nano-crystalline HEAs for use in high-strength applications without expensive refractory elements such as Mo, Ta, W, and Nb. In fact, body centered cubic NbMoTaW HEA pillar of 1 µm diameter exhibited yield strength similar to the current Al_0.1_CoCrFeNi face centered cubic nano-pillar [9]. Therefore, the current nanostructured alloy is a cost-effective alternative towards achieving ultra-strong and ductile wires for small-scale applications.

## 4. Conclusions

In conclusion, exceptional strength was seen for nano-crystalline Al_0.1_CoCrFeNi HEA similar to refractory HEAs of comparable length scales. This was attributed to grain boundary mediated plastic deformation processes. The strain rate sensitivity was higher than conventional NC material implying an even lower activation volume of dislocations.
